# Overcoming the Use of Mechanical Restraints in Psychiatry: A New Challenge in the Everyday Clinical Practice at the Time of COVID-19

**DOI:** 10.3390/jcm9113774

**Published:** 2020-11-23

**Authors:** Domenico De Berardis, Antonio Ventriglio, Michele Fornaro, Federica Vellante, Giovanni Martinotti, Silvia Fraticelli, Massimo Di Giannantonio

**Affiliations:** 1 Department of Mental Health, NHS, Psychiatric Service for Diagnosis and Treatment, Hospital “G. Mazzini”, ASL 4, 64100 Teramo, Italy; 2 Department of Neurosciences and Imaging, University “G. D’Annunzio”, 66100 Chieti, Italy; federica.vellante@gmail.com (F.V.); giovanni.martinotti@gmail.com (G.M.); silvieffesc@gmail.com (S.F.); digiannantonio@unich.it (M.D.G.); 3 Department of Psychiatry, University of Foggia, 71121, Foggia, Italy; a.ventriglio@libero.it; 4 Department of Psychiatry, Federico II University, 80131 Naples, Italy; dott.fornaro@gmail.com

Restraining interventions, which comprise physical (PR) and mechanical restraint (MR), have a long history in mental health services. PR in mental health care is defined as “…the use of physical interaction which is proposed to avoid, restrict, or subdue the normal movement of any part of the patient’s body” (Mental Health Units (Use of Force) Act, 2018). MR comprises restraining interventions that include use, control, and removal of mechanical fastening tools such as belts, anklets, and rails aimed to limit someone’s physical mobility. MR still represents a challenge and a risk for every psychiatrist who works in everyday clinical practice [1]. Although this practice is obsolete, potentially dangerous, and non-therapeutic, it is often used with a prevalence that may be different in different countries [2,3].

Some factors may be linked to MR’s increased use in patients with psychiatric and neurological disorders (Table 1). Some of them are often hard to change as they encompass environmental cues [1]. MR has generally been used in health facilities in an uncritical and unreflective mode [4]. Nevertheless, the available evidence points out that this practice is associated with several complications related to forced immobilization such as limbs injuries, pneumonia, deep venous thrombosis, and other major events comprising death due to trauma asphyxia [5,6]. It is worthy to note that the forced immobilization might also cause psychological distress and has a destructive influence on cognitive capacities and functions.

In Italy, the “Mastrogiovanni’s case” has caused a considerable stir, controversy, commotion, and media coverage [7]. The “Mastrogiovanni’s case” refers to a patient admitted in a psychiatric ward whose MR was for 84 h, the most prolonged restraint period ever reported in the literature. This MR ended with the patient’s death and caused legal consequences for almost all involved psychiatrists and nurses with several criminal convictions [7]. Even if the death of a subject during MR is an extreme and awful outcome, the use of prolonged and clumsy MR can result in several consequences and damages that, in some cases, are very severe and permanent from both physical and psychological standpoints [8,9,10]. These damages range from skin abrasions to death and demoralization to post-traumatic stress disorder (Table 1) [11,12]. Moreover, the staff applying MR might sustain physical injuries as well [13].

All considered, an intriguing and scientifically sound review entitled “Alternatives to the Use of Mechanical Restraints in the Management of Agitation or Aggressions of Psychiatric Patients: A Scoping Review” was recently published in the Journal of Clinical Medicine [14] and stimulated some considerations for us.

First, the use of MR is relatively common practice also in other medical specialties than psychiatry such as geriatrics, surgery, and internal medicine. [15]. It is frequently used in the elderly when dementia and behavioral symptoms become manifest and intractable [16,17]. Moreover, it is frequent that MR is used when the patient is confused, and the main reason is the prevention of falls [18].

Further, in surgical patients, the post-operative course may be complicated by confusion and agitation with the risk of falls. However, MR’s most common reason is to avoid taking away invasive or bothering devices [19]. PR and MR are also used in emergency departments (ED), and the Joint Commission and Centers for Medicare & Medicaid Services has realized procedures aimed at limiting the use of such procedures in US hospitals (42 CFR Part 482). Interestingly, Schnitzer et al. [20] conducted a two-year retrospective chart analysis, evaluating a sample of all adult ED visits at the Massachusetts General Hospital of Boston (USA), and found that black or African American male patients, including those without a history of violence, were at higher risk of restraint compared to white male patients. If MR is dangerous in psychiatric settings, we argue that this practice, as applied on frail subjects with several severe comorbidities, may be at greater risk of severe injuries and deaths in the setting as mentioned earlier. Thus, all efforts should be made in order to minimize this phenomenon.

Another reason for the use of MR is the problem of substance abuse and intoxication. Almost all studies on adopting MR point out the presence of acute substance abuse even in the absence of a clinically evident psychiatric disorder [21,22]. However, Martensson et al. [23] found that individuals with dual diagnoses were more frequently mechanically restrained than subjects with only psychiatric disorders or pure substance use disorder (SUD). They pointed out that, in all groups, the leading cause for MR was danger, vandalism, or assault, hence supporting the view that aggressive behaviors play a significant role in the causative pathway for it. These findings were substantially confirmed by Lykke et al. [24], who found that psychostimulant use was significantly associated with violent and aggressive behaviors and, therefore, with a higher MR risk.

On the other hand, they also noted that focused interventions might reduce the incidence of MD, thus improving treatment outcomes. Moreover, substance and alcohol abuse is frequent in subjects with personality disorder (PDs, especially antisocial and borderline personality disorders), which may further enhance the aggressive behaviors often seen in these subjects [25,26]. Kodal et al. [27] pointed out that in 82% of 114 cases of MR, the patients were diagnosed with personality disorders. Other studies also confirmed this observation that showed PDs subjects as “frequent restrained patients” [3,28,29].

Minimizing MR utilization via prevention and de-escalation should be a treatment goal for every agitated patient and trained staff [30]. Healthcare providers should conduct a careful medical assessment of all patients at risk for acute agitation. Whenever possible, begin agitation management with verbal de-escalation techniques, using pharmacologic agents to help calm the patient to strengthen the effects of verbal de-escalation [31]. It is worthy to note that often agitation might cause aggressive behaviors. Hence, definite strategies to avoid aggressive behaviors frequently try to mediate at the point of agitation. If a subject becomes agitated (revealed by behaviors like pacing, screaming, or making verbal intimidations or menacing gesticulations toward staff), the patient is usually thought to be at greater risk of aggressive behaviors and physical violence [32]. It has been demonstrated that several goals should be achieved when facing agitated patients to decrease that agitation to prevent aggressive behavior [33,34] and these include: (1) ensuring the safety of the patient, staff, and others in the area; (2) helping the patient deal with his/her feelings and anguish and keep up or recuperate control of his/her behavior, (3) avoid the use of restraining interventions if possible; and (4) avoid coercive measures that might further escalate agitation.

De-escalation skills and techniques should be taught to all healthcare workers, and staff should be trained to use them if confronted with violent or aggressive behavior. In a paper by Richmond et al. [35], the ten domains of de-escalation are as follows:

1. Always respect the personal space while maintaining a protected position with the possibility to escape in case of aggression.
2. Do not be provoking, irritating, or offensive.
3. Create verbal contact and, when possible, do not often make eye contact with the agitated patient.
4. Try to be concise and to communicate simply and clearly with the patient.
5. Recognize patients’ needs and feelings.
6. Pay close attention to what the patient is saying, without lying to them.
7. Agree or agree to disagree with the patient’s thoughts and sensations.
8. Lay down the law and set clear and definite confines.
9. Try to sincerely propose the patient’s choices and try to gain their trust.
10. Debrief the subject and the staff after the de-escalation.

The review results by Fernandez-Costa et al. [14] point out that verbal de-escalation (alone or, better, together with other techniques such as psychotherapies, the modulation of sensory stimuli, and the active participation of the patient in their own care) is always highly recommended with five reviewed articles that considered it to be the first-choice intervention. We agree with this observation, and we firmly believe that short-duration MR should be used only as a last resort in subjects who continue to remain a danger to themselves and/or others when verbal de-escalation and use of medication fail. Moreover, in such cases, all efforts must be made to remove the patient from restraints as soon as possible [36]. The timely and rational pharmacological therapies with parenteral benzodiazepines and antipsychotics would also help calm the subject and reduce the length of MR and must be evaluated [37,38].

However, to reduce the use of MR, it has been also demonstrated that a positive attitude in not using MR, together with staff training, working cohesion, and healthcare workers formation, may be beneficial in the recourse to MR [39]. It is imperative and crucial to improve knowledge and adequately train hospital staff in psychiatric wards, but also in all wards potentially involved in the use of MR, with appropriate methods [40,41]. Staff training and selection are an effective measure to minimize MR’s duration and adverse effects, and we recommend conducting a compulsory training program to reduce unnecessary restraint [42,43].

The results of the review by Fernandez-Costa et al. [14] are an important landmark in the prevention and reduction in MR’s use and can be the basis of useful knowledge of this topic. We totally agree with them when they write, “The approach towards an agitated or violent patient should begin with less coercive measures”.

## Figures and Tables

**Table 1:** Factors and potential consequences associated with mechanical restraint (MR) in patients with psychiatric and neurological disorders.

Factors Commonly Associated with Higher Likelihood of MR	Potential Consequences of MR
Male gender	Increased agitation if MR is prolonged
Poor socioeconomic condition and/or homelessness	Symptoms’ worsening if MR is prolonged
Younger or elderly people	Physical injuries if MR is clumsy or prolonged
Involuntary admission	Perceptions of being physically and mentally abused or treated like criminals
Multiple previous inpatient admissions	Strong feelings of humiliation
Psychomotor agitation with violence	Loss of trust in staff
History of aggression	Reduced adherence to treatment if MR is prolonged
Staff-directed assault	Decreased self-esteem and empowerment
Self-harming behaviors	Increased perception of helplessness if MR is prolonged
Substance abuse or intoxication (especially alcohol and psychostimulants)	Post-traumatic stress disorder
Schizophrenia and other psychotic disorders	Potential negative impact on healthcare professionals directly involved in restraining patients
Personality disorders (antisocial personality disorder, borderline personality disorder)	Permanent lesions (on wrists, legs, nerves, etc.)
Delirium	Death (venous thromboembolism, deep vein thrombosis, pulmonary embolism, mechanical asphyxia, aspiration and breathing difficulties, stress cardiomyopathy, drug-induced liver injury)
Neurodegenerative disorders	
History of epilepsy	
